# Building the capacity of West African countries in *Aedes* surveillance: inaugural meeting of the West African *Aedes* Surveillance Network (WAASuN)

**DOI:** 10.1186/s13071-022-05507-0

**Published:** 2022-10-21

**Authors:** Samuel K. Dadzie, Jewelna Akorli, Mamadou B. Coulibaly, Koffi Mensah Ahadji-Dabla, Ibrahima Baber, Thierry Bobanga, Ali Ould Mohamed Salem Boukhary, Tiago Canelas, Luca Facchinelli, Adéritow Gonçalves, Moussa Guelbeogo, Basile Kamgang, Ibrahima Kalil Keita, Lucien Konan, Rebecca Levine, Nicole Dzuris, Audrey Lenhart, WAASuN contributors, Maxwell Appawu, Dogunro Festus Ayorinde, Daniel Boakye, Mawlouth Diallo, João Dinis, John Soleemulo Fayiah, Boube Hamani, Eloy Emelda Idam, Cani Pedro Jorge, Balla Kandeh, Olakiigbe Abiodum Kanmi, Raphael N’Guessan, Sellase Pi-Bansa, Sidina Mohamed Salem, Ansumana Sillah, Samuel Smith, Hyacinthe Toé, Chrispin Williams, Michael Wilson, Anges Yadouleton

**Affiliations:** 1 Department of Parasitology, Noguchi Memorial Institute for Medical Research, University of Ghana, Accra, Ghana; 2 University of Sciences, Techniques and Technologies of Bamako, Bamako, Mali; 3 Department of Zoology, Faculty of Sciences, University of Lomé, Lomé, Togo; 4 Abt Associates, US President’s Malaria Initiative (PMI) VectorLink Project, Monrovia, Liberia; 5 Services de Parasitologie et d’Entomologie, Département de Médecine Tropicale, Faculté de Médecine, Université de Kinshasa, Kinshasa, Democratic Republic of the Congo; 6 Université de Nouakchott Al Aasriya, Nouakchott, Mauritania; 7 Medical Research Council Epidemiology Unit, University of Cambridge, Cambridge, UK; 8 Liverpool School of Tropical Medicine, Liverpool, UK; 9 Laboratory of Medical Entomology, National Institute of Public Health, Praia, Cape Verde; 10 Centre National de Recherche et de Formation sur le Paludisme and University Joseph Ki-Zerbo, Ouagadougou, Burkina Faso; 11 Department of Medical Entomology, Centre for Research in Infectious Diseases, Yaounde, Cameroon; 12 National Malaria Control Program, Conakry, Guinea; 13 Department of Malaria and Emerging Disease, National Institute of Public Hygiene, Abidjan, Côte d’Ivoire; 14 US Centers for Disease Control and Prevention, Atlanta, USA

## Abstract

Arboviral diseases such as dengue, Zika and chikungunya transmitted by *Aedes* mosquitoes have been reported in 34 African countries. Available data indicate that in recent years there have been dengue and chikungunya outbreaks in the West Africa subregion, in countries including Côte d’Ivoire, Burkina Faso, Gabon, Senegal, and Benin. These viral diseases are causing an increased public health burden, which impedes poverty reduction and sustainable development. *Aedes* surveillance and control capacity, which are key to reducing the prevalence of arboviral infections, need to be strengthened in West Africa, to provide information essential for the formulation of effective vector control strategies and the prediction of arboviral disease outbreaks. In line with these objectives, the West African *Aedes* Surveillance Network (WAASuN) was created in 2017 at a meeting held in Sierra Leone comprising African scientists working on *Aedes* mosquitoes. This manuscript describes the proceedings and discusses key highlights of the meeting.

**Graphical Abstract:**

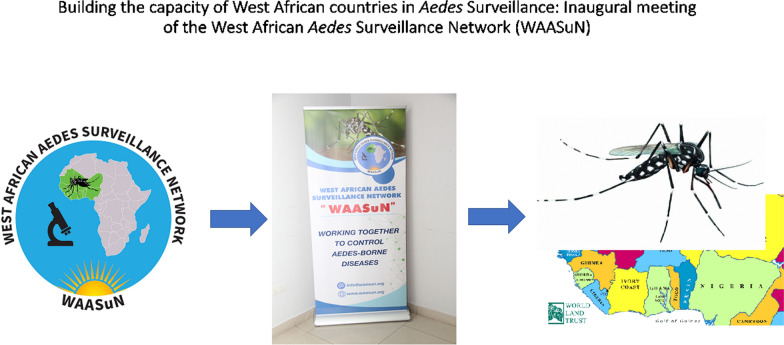

## Meeting report

*Aedes*-borne arboviral diseases such as dengue, Zika and chikungunya are being reported with increasing frequency across Africa, and despite being vaccine preventable, yellow fever outbreaks continue to persist [1–3]. In sub-Saharan Africa, where both the domesticated *Aedes aegypti aegypti* and the sylvatic *Aedes aegypti formosus* subspecies originated and coexist [4], the epidemiology of mosquito-transmitted viruses is less clear as compared to the rest of the world. In the last 50 years, studies on the biology of *Ae. aegypti* have been primarily performed on the domestic form *Ae. aegypti aegypti* outside the African continent, focusing on its role as a vector and its interactions with humans in urban and semi-urban environments. Although Africa was the cradle of modern *Aedes* surveillance and control methodologies stemming from the yellow fever research activities undertaken in the early twentieth century, current entomological capacity is primarily focused on malaria vectors. This has resulted in a tremendous knowledge gap on arbovirus epidemiology in sub-Saharan Africa, whereby most countries lack routine surveillance programs, trained personnel, and control activities that are focused on *Aedes* and the viruses they transmit [5]. As outbreaks of *Aedes*-borne arboviruses continue to increase across Africa, establishing a strong public health entomology infrastructure around *Aedes* mosquitoes is critical to both containing and preventing outbreaks.

Given recurrent yellow fever outbreaks and the increasing public health burden due to dengue and chikungunya, West Africa is a priority region for strengthening public health entomology capacities around *Aedes* surveillance and control [5]. The idea for the West African *Aedes* Surveillance Network (WAASuN) arose in 2017 at a meeting held in Freetown, Sierra Leone, comprising African scientists working on various aspects related to the biology, control, and surveillance of *Aedes* mosquitoes. WAASuN aims to strengthen the capacity of West African countries to understand the biology of *Aedes* mosquitoes and define their roles as vectors, carry out surveillance and control of *Aedes* arboviral disease vectors, and facilitate collaboration between countries and other partners on various aspects of *Aedes* surveillance and control. WAASuN is now a non-profit organization registered in Ghana. The network’s secretariat was formally established at the Noguchi Memorial Medical Institute in Accra, Ghana in July 2019, as part of the inaugural meeting described in this manuscript. The specific objectives of the network include (i) strengthening existing linkages in *Aedes* surveillance both within and between countries, (ii) building the capacity of member countries in surveillance activities and outbreak preparedness, and (iii) advocating for increased investment in *Aedes* surveillance activities in member countries. The network currently has a membership of 60 scientists from West Africa and other partner countries. Herein, we describe the proceedings of the inaugural meeting of WAASuN, held from 8 to 12 July 2019 in Accra, Ghana.

**Fig. 1:**
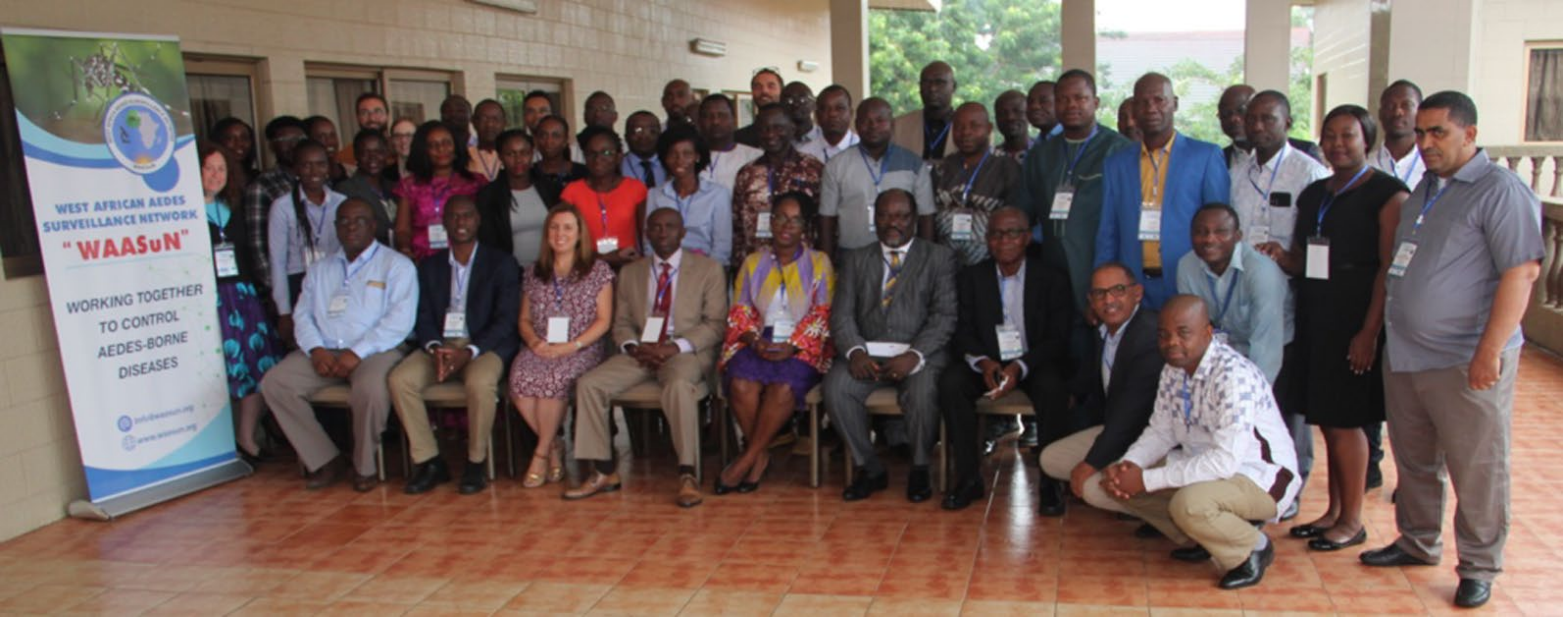
Participants at the West African *Aedes* Surveillance Network meeting in Accra, Ghana

The meeting was opened with welcoming addresses and opening remarks by WAASuN co-chairs Dr. Samuel Dadzie and Dr. Mamadou Coulibaly. Dr. Dadzie presented the objectives of the meeting, which were to (i) receive updates from countries on *Aedes* surveillance activities, (ii) receive information from other partners on *Aedes* research and surveillance activities and protocols, (iii) share updates on WAASuN activities and the development of the terms of reference and strategic plan, (iv) participate in a hands-on capacity building activity on the laboratory detection of *kdr* mutations in *Ae. aegypti*, and (v) network with other attendees (Fig. 1).

The participants gave presentations that summarized the current *Aedes* surveillance and control activities in their countries. The following countries were represented: Angola, Benin, Burkina Faso, Cameroon, Cape Verde, Cote d’Ivoire, Democratic Republic of Congo, Gambia, Ghana, Guinea, Guinea-Bissau, Liberia, Mali, Mauritania, Niger, Nigeria, Senegal, Sierra Leone, and Togo.

Presentations and updates were also provided from partners, including the Liverpool School of Tropical Medicine and the Partnership for Increasing the Impact of Vector Control, which aims to reduce the burden of vector-borne diseases by increasing the research capacity of African and UK scientists and building partnerships to develop sustainable vector control solutions.

The subsequent session reviewed the highlights and takeaways from the preceding country and partner presentations. It was agreed that countries need to harness resources and/or work in conjunction with successful programs with strong entomology components such as national malaria control programs and the US President’s Malaria Initiative to conduct *Aedes* surveillance. For example, rather than discarding the *Aedes* inadvertently collected during routine *Anopheles* surveillance, a small amount of funding could allow for the processing of these samples. Through further networking in the region, capacity can be enhanced; countries with limited surveillance capacity could be supported by countries with more advanced surveillance activities. Additionally, a One Health approach for arboviral disease outbreaks is important, as many are zoonotic, and insecticide deployment should be informed by resistance profiles.

It was also highlighted that many countries are experiencing similar challenges. There is a common lack of efficient and standardized tools for *Aedes* collection. There is also a lack of sensitive indices to determine *Aedes* densities that correlate to disease transmission risk. Insecticide resistance threatens the success of existing control strategies, and assays using insecticide concentrations meant for detecting resistance in *Anopheles* may not be appropriate for *Aedes*. Misdiagnosing arboviral diseases as malaria was also a common challenge, as arboviral diseases have similar symptoms and manifestations. Additionally, the lack of funding for *Aedes* surveillance is a common challenge to initiating, strengthening, and maintaining programs.

Key points that had been raised during the country presentations were discussed. The first topic was lorry tires, with the consensus that a legal framework to enforce regulations around tire disposal and storage would be needed to eliminate them as a key source of *Aedes*. In addition, the private sector could be engaged to potentially recycle tires for use as chairs, shoes, or other commodities.

The next point of discussion centered around suitable collection methods for *Aedes.* It was determined that the collection method must consider key objectives such as the species, mosquito behavior, feeding preferences, etc., and that one trap type may not be suitable for all settings. Several traps could be tested in different countries and data collected for standardization of methods. The group generally agreed that ovitraps baited with infusions work best for egg collection, BG-Sentinel traps work well for collecting both indoor and outdoor host-seeking adult *Aedes*, gravid traps are preferred for collecting gravid *Aedes* outdoors, and US Centers for Disease Control and Prevention (CDC) light traps and CDC ultraviolet traps are preferred for sylvatic *Aedes* species. It was agreed that Prokopack aspirators work well for indoor resting mosquito collections. Upon collecting *Aedes*, another common challenge is distinguishing between species; although morphological keys exist, they are not available for all species. Microscopy and molecular assays can help distinguish species, but equipment and supplies are often lacking.

Several country presentations included data on immature indices, but since these are not predictive of disease, some participants questioned their use. It was discussed that immature indices are useful for estimating densities, locating infestations for control efforts, and predicting areas of high risk for arboviruses. One key gap identified is the lack of formal *Aedes* surveillance activities in countries.

Another area of discussion was insecticide resistance testing in *Aedes*. Diagnostic doses for *Aedes* have not been recommended for many insecticides, so countries often use the same doses as for *Anopheles*. Different countries use different susceptible *Ae. aegypti* strains such as Bora Bora or New Orleans, but ideally a susceptible African strain (such as the one maintained by Benin) should be used for reference. Also, a large focus has been on phenotypic resistance, but it is also important to document mechanisms of resistance, and this was identified as a gap. Issues of heterogeneity of *Aedes* resistance mechanisms on spatial and temporal scales were discussed. For *Aedes*, it is not ideal to extrapolate results to a wider area when samples from only a few sites are tested.

Capacity building was another important point of discussion. Some ideas to improve capacity building on a regional scale included collaborating with the West African Health Organization, and multiple countries jointly applying for grants to help mobilize funding. In this way, a consortium could be formed to embark on projects through which member countries could be trained.

The meeting also contained elements of capacity strengthening. Participants were introduced to the CDC Epi Info Vector mobile surveillance system, which can be used to enter entomological data directly in the field, as an alternative to collecting data using paper forms. An additional component of the meeting was dedicated to insecticide resistance, including an overview of the genetics and dynamics of insecticide resistance in *Ae. aegypti*. Participants also participated in a laboratory practical of real-time polymerase chain reaction to detect the F1534C, V1016I, and V410L *kdr* mutations associated with insecticide resistance in *Ae. aegypti*.

The meeting also provided an opportunity to discuss and finalize the terms of reference and strategic plan for WAASuN. The discussions on the strategic plan were carried out within the framework of the objectives of the network, which include (i) strengthening existing linkages in *Aedes* surveillance both within and between countries, (ii) bridging the gap between existing surveillance systems in the subregion, (iii) strengthening the capacity of member countries in surveillance activities and outbreak preparedness, and (iv) advocating for funding of *Aedes* surveillance activities in member countries. At the end of the discussions, members agreed on a 5-year key activity plan for the network, including (i) planning and hosting of the next meeting in Côte d’Ivoire, (ii) resource mobilization through payment of dues and proposal writing, (iii) community education activities, and (iv) capacity building in various aspects of *Aedes* surveillance and control within member countries.

The meeting closed with a discussion of the objectives to accomplish before the next WAASuN meeting, which had been planned for Côte d’Ivoire in July 2020 but was postponed until 2022 due to COVID-19. These objectives included registering WAASuN as a non-governmental organization in Ghana, finalizing the terms of reference and strategic plan and circulating them among members, updating membership terms and dues, and preparing the proceedings of this meeting for publication. Since the meeting, WAASuN members have continued to provide leadership on *Aedes* surveillance in West Africa and have applied for funding opportunities. Key members of the network have been instrumental in supporting the World Health Organization’s Special Programme for Research and Training in Tropical Diseases in the drafting of the *Aedes* surveillance entomological protocol, which is currently being piloted in some West African countries. Members of the network have also been providing support to the West African Health Organization and the World Health Organization’s Special Programme for Research and Training in Tropical Diseases by building the entomological capacity of seven West African countries, Niger, Nigeria, Cape Verde, Mauritania, Burkina Faso, Senegal and Côte d’Ivoire, in *Aedes* vector surveillance. In conclusion, the inaugural meeting of WAASuN solidified a structure and mechanism for West African countries to increase focus on *Aedes* surveillance activities in preparedness for future outbreaks of arboviral diseases.
